# Experimental Determination of the Membrane Topology of the *Plasmodium* Protease Plasmepsin V

**DOI:** 10.1371/journal.pone.0121786

**Published:** 2015-04-07

**Authors:** Sarah J. Tarr, Andrew R. Osborne

**Affiliations:** Institute of Structural and Molecular Biology, Division of Biosciences, Birkbeck and University College London, London, United Kingdom; Institut national de la santé et de la recherche médicale—Institut Cochin, FRANCE

## Abstract

The malaria parasite exports hundreds of proteins into its host cell. The majority of exported proteins contain a Host-Targeting motif (also known as a *Plasmodium* export element) that directs them for export. Prior to export, the Host-Targeting motif is cleaved by the endoplasmic reticulum-resident protease Plasmepsin V and the newly generated N-terminus is N-α-acetylated by an unidentified enzyme. The cleaved, N-α-acetylated protein is trafficked to the parasitophorous vacuole, where it is translocated across the vacuole membrane. It is clear that cleavage and N-α-acetylation of the Host-Targeting motif occur at the endoplasmic reticulum, and it has been proposed that Host-Targeting motif cleavage and N-α-acetylation occur either on the luminal or cytosolic side of the endoplasmic reticulum membrane. Here, we use self-associating ‘split’ fragments of GFP to determine the topology of Plasmepsin V in the endoplasmic reticulum membrane; we show that the catalytic protease domain of Plasmepsin V faces the endoplasmic reticulum lumen. These data support a model in which the Host-Targeting motif is cleaved and N-α-acetylated in the endoplasmic reticulum lumen. Furthermore, these findings suggest that cytosolic N-α-acetyltransferases are unlikely to be candidates for the N-α-acetyltransferase of Host-Targeting motif-containing exported proteins.

## Introduction

Malaria is caused by eukaryotic parasites of the genus *Plasmodium*. In 2010, there were over 200 million cases of the disease, of which approximately 655 000 were fatal [1]. Symptoms of infection occur during the blood stage of the parasite lifecycle, when the parasites invade and replicate within erythrocytes. Inside the erythrocyte, the parasite resides within a vacuole known as the parasitophorous vacuole (PV). PV-resident proteins synthesised within the parasite are transported to the PV via the secretory pathway. The parasite also exports many proteins across the PV membrane into the host cell. Exported proteins cause alterations to the erythrocyte that are required for virulence [2]. Many exported proteins and proteins involved in the export pathway are essential for parasite survival [2–4].

Many exported proteins are directed for export across the PV membrane by a Host-Targeting (HT) motif, also known as a *Plasmodium* export element, which has the consensus Arg-X-Leu-X-Glu/Asp/Gln (where X is any amino acid) [5–7]. The HT motif sits approximately 30 residues downstream of an N-terminal transmembrane domain that is required to direct the exported proteins into the endoplasmic reticulum (ER) [8]. Prior to export, the HT motif is cleaved after the Leu residue [5], by the ER-resident, membrane-anchored protease Plasmepsin V [9,10]. The newly generated N-terminus is then N-α-acetylated [5,11]. In the simplest model for protein export, these modifications would occur in the lumen of the endoplasmic reticulum [9,10,12]. The cleaved, N-α-acetylated protein is then trafficked to the PV via the secretory pathway [11,13–16], where it is translocated across the PV membrane into the host cell.

The significance of N-α-acetylation of the new N-terminus generated upon cleavage of the HT motif by Plasmepsin V is not clear, and the identity of the responsible N-α-acetyltransferase is not known. Typically, N-α-acetylation is a modification limited to cytosolic proteins [17,18]. This has led to the proposal of an alternative model for protein export in which HT motif cleavage by Plasmepsin V and N-α-acetylation of the new N-terminus of exported proteins occur in the cytosol [19], and that the acetyltransferase of exported proteins may belong to the cytosolic NatA-D family. A model where HT motif cleavage and subsequent N-α-acetylation occur on the cytosolic side of the ER membrane would also require localisation of the catalytic domain of Plasmepsin V in the cytosol.

**Fig 1:**
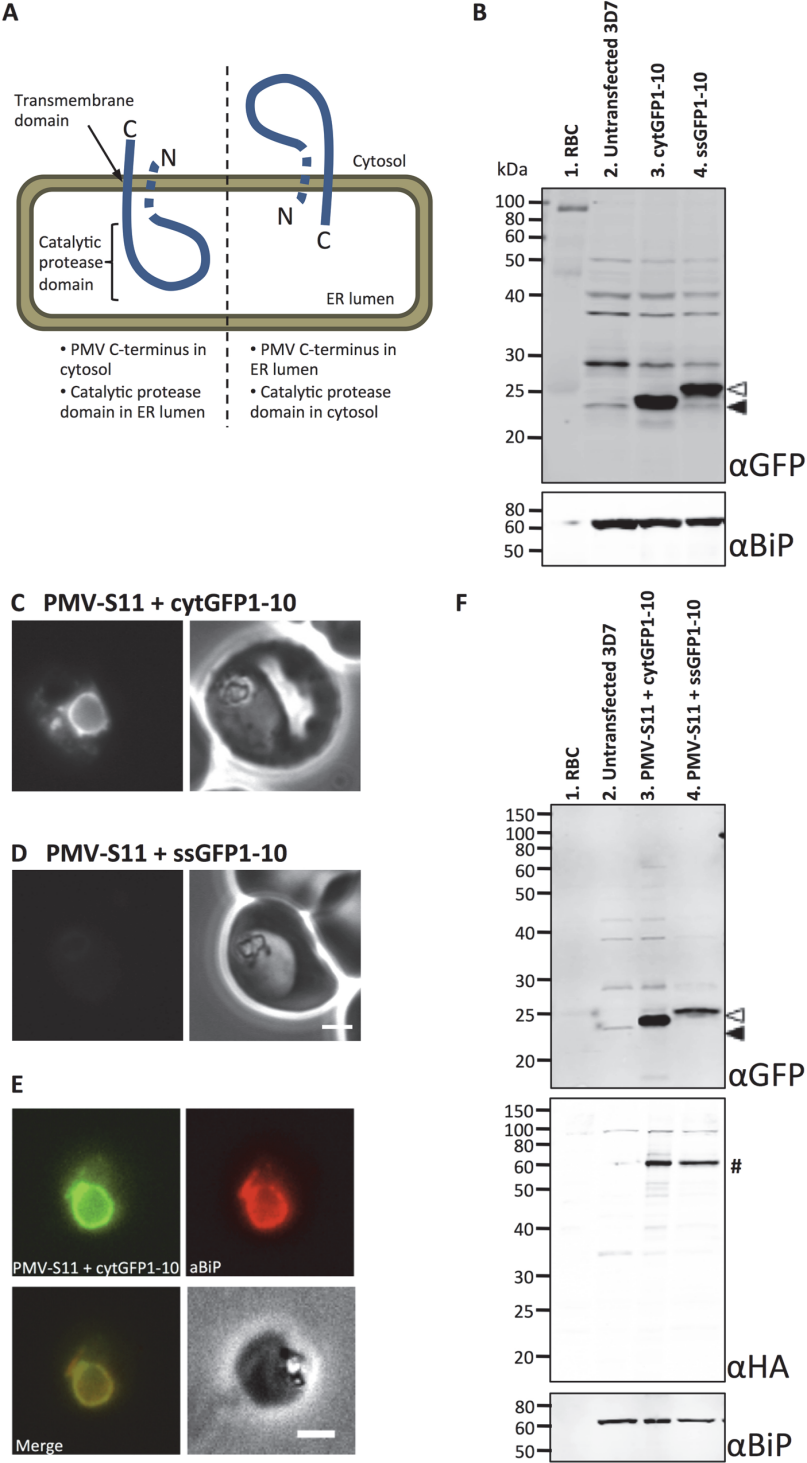
Using split GFP to establish the topology of Plasmepsin V. (A) Possible topologies of PMV in the ER membrane. It remains to be established whether the hydrophobic signal sequence (or possibly transmembrane domain) at the N-terminus of PMV is present or cleaved in the mature protein (depicted as a dashed region) [20]. Left: C-terminus of PMV in the cytosol would locate the catalytic protease domain in the ER lumen; Right: C-terminus of PMV in the ER lumen would locate the catalytic protease domain in the cytosol. (B) Immunoblots of schizont preparations from transfected parasite lines. 5 x 10^5^ schizonts were loaded per lane. Top: anti-GFP; Bottom: loading control, anti-BiP. Lane 1: Uninfected red bloods cells; Lane 2: Untransfected *P*. *falciparum* 3D7; Lane 3: cytGFP1-10 (filled arrow); Lane 4: ssGFP1-10 (unfilled arrow). (C-D) Fluorescence (left) and phase contrast (right) images of *P*. *falciparum* parasites expressing PMV-S11 with cytGFP1-10 (C) and PMV-S11 with ssGFP1-10 (D). Scale bar, 2 m. (E) Immunofluorescence colocalisation of the ER marker BiP (red) with the GFP fluorescence (green) in parasites expressing PMV-S11 with cytGFP 1–10. Scale bar, 2 m. Additional representative images are shown in S3 Fig (F) Immunoblots of schizont preparations from PMV construct-transfected parasite lines. 5 x 10^5^ schizonts were loaded per lane. Top: anti-GFP; Middle: anti-HA; Bottom: loading control, anti-BiP. Lane 1: Uninfected red bloods cells; Lane 2: Untransfected *P*. *falciparum* 3D7; Lane 3: PMV-S11 (hash) co-expressed with cytGFP1-10 (filled arrow); Lane 4: PMV-S11 (hash) co-expressed with ssGFP1-10 (unfilled arrow).

Plasmepsin V is anchored in the parasite ER membrane by a transmembrane domain at its C-terminus [9,20]. This domain lies downstream of the central, catalytic protease domain. Consequently, the catalytic domain and the C-terminus of Plasmepsin V lie on opposite sides of the ER membrane. Transmembrane domains can integrate into the ER membrane in two possible orientations; with the C-terminus either on the cytoplasmic or luminal side of the ER membrane. Therefore, Plasmepsin V has two potential topologies in the ER membrane, namely with its C-terminus in the cytosol or in the ER lumen (Fig 1A).

Membrane protein topology can be determined experimentally by tagging proteins of interest with topology reporters [21–23]. One example is the use of self-associating fragments of superfolder green fluorescent protein (GFP), or ‘split’ GFP. GFP forms an eleven-stranded beta barrel. Split GFP can be expressed as two separate polypeptide chains, comprising beta strands 1–10 (GFP1-10) and beta strand 11 (S11). Alone, neither polypeptide is fluorescent but if the two fragments are either added together *in vitro* or co-expressed *in vivo*, they can associate to reconstitute a functional fluorescent complex [24]. Split GFP has been used to study protein localisation, interactions, and solubility [25]. By directing GFP1-10 to known cellular compartments, this technique can reveal the topology of transmembrane proteins C-terminally tagged with the S11 fragment, since the formation of a fluorescent complex is dependent on the C-terminus of a membrane protein residing in the same cellular compartment as the GFP1-10 fragment [22]. In *Plasmodium*, the topology of the ER protein Der1-1 was established using this method [23].

Here, we have used split GFP to determine the topology of the ER protease, Plasmepsin V, in *Plasmodium falciparum*. By expressing Plasmepsin V with a C-terminal S11 tag in parasites that express GFP1-10 that is targeted either to the parasite cytoplasm or to the secretory pathway, we are able to show that Plasmepsin V is orientated with its C-terminus in the parasite cytosol; this strongly supports a model in which the catalytic domain of Plasmepsin V is in the ER lumen. These data confirm that the new N-terminus created by Plasmepsin V cleavage is generated within the ER lumen. Consequently, it is very unlikely that the N-α-acetyltransferase of HT motif-containing exported proteins is a cytosolic enzyme.

## Materials and Methods

### Ethics statement

The experiments described here were approved by the National Health Service National Research Ethics Service, UK, East London REC3 committee (Research Ethics Committee reference 10H/H0701/121).

### Plasmids and parasite transfection

DNA encoding *P*. *falciparum* proteins was PCR amplified from *P*. *falciparum* 3D7 genomic DNA and cloned into a *P*. *falciparum* expression plasmid, in frame with 3’ single- or triple-hemagglutinin and S11 or eGFP tags. *P*. *falciparum* expression plasmid backbones were similar to those described in Tarr et al. (2013) [26]; expression of GFP1-10-, S11- and eGFP-tagged proteins was under the control of PfCAM 5’ and PbDT 3’ regions; expression of selectable markers was under the control of PcDT 5’ and HrpII 3’ regions. Constructs were transfected into *P*. *falciparum* 3D7 parasites with pHTH [27] as described previously [28]. The S11-tagged and eGFP-tagged constructs contained a human dihydrofolate reductase selection cassette (DHFR) and were selected with 2.5 nM WR99210 (Jacobus Pharmaceutical Company). The cytGFP1-10 and ssGFP1-10 constructs contained a blasticidin deaminase selection cassette and were selected with 2 μg/ml Blasticidin (Invivogen).

DNA encoding maltose-binding protein was PCR amplified from pMAL-4E (NEB). Plasmids containing GFP1-10 and S11 fragments were a gift from Geoffrey Waldo.

### Microscopy

For live cell microscopy, mixed-stage parasites were imaged one day after feeding by placing a drop of culture material between a microscope slide and cover slip. Fluorescence and phase contrast images were acquired using a Zeiss Axiovert S100 microscope, equipped with a HBO100 lamp, an FDI Photonic Sciences camera, and ImagePro Plus software. Images of parasite lines expressing split GFP fragments were acquired using identical exposure times. Identical brightness and contrast settings were applied to images using ImageJ.

For immunofluorescence microscopy, fluorescence and phase contrast images were acquired using a Zeiss Axiovert 200 M microscope, equipped with a HBO100 lamp and Axiovision software. Images were processed in ImageJ and adjusted using automatic brightness and contrast settings.

### Immunofluorescence staining

Infected red blood cells were adhered to polyethyleneimine-coated cover slides and fixed in 4% paraformaldehyde in phosphate-buffered saline (PBS) for two hours at room temperature. The fixative was quenched in 50 mM Tris pH 8, 150 mM NaCl for 15 minutes. Cells were then permeabilised in 0.5% Triton X-100 and blocked 0.5% Triton X-100 with 1% fish skin gelatin (FSG). Subsequent washes and antibody incubations were performed in 0.1% Triton X-100 in PBS with 1% FSG; cells were labelled with an anti-BiP antibody (a gift from Anthony Holder) and Alexa 594-labelled secondary antibody (Molecular Probes).

### Protein detection by immunoblotting

Expression of proteins in transfected parasite lines was confirmed by SDS-PAGE and immunoblotting of purified schizonts. 5 x 10^5^ schizonts were loaded per lane. Immunoblots were probed with rabbit anti-GFP antibody (Torrey-Pines), mouse anti-HA antibody (Invitrogen) and a rabbit anti-BiP antibody (a gift from Jude Przyborski). Immunoblots were imaged using a LI-COR Odyssey imager.

## Results

### Expression of split GFP fragments in the *Plasmodium* cytosol and secretory pathway

To use split GFP as to tool to probe the topology of Plasmepsin V, *Plasmodium* expression constructs were generated that localised the GFP1-10 fragment to the parasite cytosol or the endoplasmic reticulum lumen. These are referred to as cytGFP1-10 that lacks any targeting information and ssGFP1-10 that is fused to the signal sequence of BiP, respectively. When cytGFP1-10 and ssGFP1-10 were expressed in parasites, no fluorescence was detectable as would be expected given the incomplete nature of the GFP β-barrel lacking the 11^th^ β-strand (cytGFP1-10, S1 Fig, panel A; ssGFP1-10, S1 Fig, panel B). Expression of both proteins was confirmed by immunoblotting of purified schizonts with an anti-GFP antibody (Fig 1B, top blot, lane 3 filled arrow and lane 4 unfilled arrow, respectively). As seen previously [23], cytGFP1-10 and ssGFP1-10, when co-expressed with an S11 tagged protein in the same cellular compartment could form a fluorescent complex (see S1 Text).

### Determination of the topology of Plasmepsin V using split GFP

A construct encoding Plasmepsin V tagged at the C-terminus with S11 (PMV-S11) was expressed in *P*. *falciparum* co-expressing either cytGFP1-10 or ssGFP1-10. A triple HA tag was also included between PMV and the S11 tag for immunodetection. Fluorescent parasites were observed when PMV-S11 was co-expressed with cytGFP1-10 (Fig 1C), but not when co-expressed with ssGFP1-10 (Fig 1D). In immunoblots of schizont preparations from both of the PMV-S11 co-transfected lines, bands of approximately 65 kDa were detectable using an anti-HA antibody, as expected for intact PMV-S11 (predicted molecular weight, 74.6 kDa; Fig 1F, middle blot, lanes 3 and 4, marked by a hash). The cytGFP1-10 and ssGFP1-10 proteins in PMV-S11 co-transfected parasites were detectable using an anti-GFP antibody (Fig 1F, top blot, lane 3 filled arrow and lane 4 unfilled arrow, respectively).

ER localisation of the PMV-S11- cytGFP1-10 complex was confirmed by immunofluorescence analysis; the GFP signal in parasites expressing PMV-S11 with cytGFP1-10 showed extensive colocalisation with the ER marker BiP (Fig 1E). This confirms that the PMV-S11- cytGFP1-10 complex was localised to the ER.

To ensure that the ssGFP1-10 is functional we confirmed the topology of the C-terminal transmembrane domain of *Plasmodium falciparum* Equilibrative Nucleoside Transporter 1 (PfENT1; PF3D7_1347200), which has its C-terminus located in the secretory pathway (see S1 Text).

Together these data confirm that the C-terminus of Plasmepsin V resides within the parasite cytosol and consequently that the catalytic protease domain is situated within the ER lumen.

## Discussion

Many exported proteins contain an N-terminal transmembrane domain that directs entry of the nascent exported protein into the endoplasmic reticulum [29], and a Host-Targeting motif that directs them for export across the PV membrane into the host erythrocyte [6,7]. Prior to export, the HT motif is cleaved at the ER by the transmembrane protease Plasmepsin V to generate a new N-terminus that is N-α-acetylated by an unknown enzyme. In the simplest model for protein export, HT motif cleavage and N-α-acetylation occur in the lumen of the endoplasmic reticulum [9,10]. However, since N-α-acetylation is primarily a cytosolic modification, a second model has been proposed in which cleavage and N-α-acetylation occur on the cytosolic side of the ER membrane [19].

Here, we have determined the topology of Plasmepsin V in the ER membrane. We have shown that the C-terminus of Plasmepsin V lies on the cytosolic side of the ER membrane, and consequently that the catalytic protease domain likely resides within the ER lumen. Therefore, our data support the model for protein export in which cleavage of the HT motif occurs in the lumen of the ER. These data are also consistent with biochemical analysis of purified Plasmepsin V that indicated that the enzyme is maximally active at a pH range more consistent with an environment such as the ER lumen than the cytosol [9]. It has also been shown that a protein export reporter lacking an N-terminal transmembrane domain but retaining an intact HT motif accumulated in the parasite cytosol and showed no evidence of HT motif cleavage [11]. This indicates that there is no Plasmepsin V activity in the cytosol, and therefore that the topology we describe here is indeed the functional topology of the enzyme.

The N-α-acetyltransferase of HT motif-containing exported proteins has not been identified. Our data indicate that the substrate for N-α-acetylation of HT motif-containing exported proteins is generated by Plasmepsin V in the lumen of the ER. The N-α-acetyltransferase of HT motif-containing exported proteins is therefore also likely to reside within this compartment, rather than being a cytosolic enzyme. The contribution of the N-α-acetylation modification to protein export is not known; the N-α-acetylated, cleaved HT motif is necessary and sufficient to signal protein export [26,30] but an N-α-acetylated N-terminus located in the PV lumen is alone not sufficient for efficient protein export [5,26]. If N-α-acetylation is a necessary for export, for example to protect the N-terminus of mature exported proteins from degradation, the unidentified N-α-acetyltransferase of HT motif-containing exported proteins may represent a novel drug target. N-α-acetyltransferases are primarily cytosolic enzymes associated with a range of functions [31]. Therefore, an N-α-acetyltransferase that functions within the secretory pathway may have divergent characteristics to N-α-acetyltransferases that are active in the cytosol, making it a good potential candidate for therapeutics.

*Plasmodium* parasites also export a class of exported proteins known as PEXEL-negative exported proteins (PNEPs). PNEPs include the virulence-associated *P*. *falciparum* erythrocyte membrane protein 1 family, as well as other virulence-associated proteins [32–35]. PNEPs are trafficked via the ER and follow a similar export pathway to HT-motif containing proteins [3,4,30] but lack an HT motif and are not processed by Plasmepsin V. The N-terminal sequences of many PNEPs contain information signaling export and in some cases are found to be proteolytically processed [36], but do not contribute to translocation into the ER, which is initiated by internal transmembrane domain(s) [37]. Models for how the export signal of PNEPs is generated differ to those of HT-motif containing proteins, as the N-terminal sequences of nascent PNEPs do not enter the ER co-translationally until an internal transmembrane domain is translated. In the time between synthesis of the N-terminal residues of a PNEP and synthesis of the internal transmembrane domain(s), the N-terminal residues are exposed to the parasite cytosol, where it is likely that methionine amino peptidase removes the N-terminal methionine and the N-terminus may be cleaved by an unidentified protease(s) [36]. In this case, the N-termini of PNEPs may also be substrates for cytosolic N-α-acetyltransferases.

Understanding the mechanisms of protein export by the malaria parasite is crucial if this essential pathway is to be considered a target for the development of antimalarial therapeutics. By confirming the topology of a key enzyme in the export pathway of HT motif-containing proteins, we have defined the subcellular environment in which critical steps in the export of HT motif-containing proteins occur. These results should guide the identification of as yet uncharacterised components of the HT motif-dependent export pathway.

## Supporting Information

S1 Fig S11 split GFP fragments associate to form a fluorescent complex with cytGFP1-10 and ssGFP1-10. (A-B) Fluorescence (left) and phase contrast (right) images of *P*. *falciparum* parasites expressing cytGFP1-10 (A) and ssGFP1-10 (B). (C) Immunoblots of schizont preparations from transfected parasite lines. 5 x 10^5^ schizonts were loaded per lane. Top: anti-GFP; Middle: anti-HA; Bottom: loading control, anti-BiP. Lane 1: Uninfected red bloods cells; Lane 2: Untransfected *P*. *falciparum* 3D7; Lane 3: cytGFP1-10 (filled arrow); Lane 4: ssGFP1-10 (unfilled arrow); Lane 5: cytMBP-S11 with cytGFP1-10; Lane 6: cytMBP-S11 with ssGFP1-10; Lane 7: ssMBP-S11 with cytGFP1-10; Lane 8: ssMBP-S11 with ssGFP1-10; Lane 9: Uninfected red bloods cells; Lane 10: Untransfected *P*. *falciparum* 3D7; Lane 11: cytMBP-eGFP; Lane 12: ssMBP-eGFP. CytMBP-S11 and ssMBP-S11 are marked with a hash, and cytMBP-eGFP and ssMBP-eGFP are marked with an asterisk. CytMBP-eGFP migrated as a doublet. Lanes 1–4 are also depicted in Fig 1B. (D-I) Fluorescence (left) and phase contrast (right) images of *P*. *falciparum* parasites expressing cytMBP-S11 with cytGFP1-10 (D); cytMBP-eGFP (E); cytMBP-S11 with ssGFP1-10 (F); ssMBP-S11 with cytGFP1-10 (G); ssMBP-S11 with ssGFP1-10 (H) and ssMBP-eGFP (I). Scale bar, 2 m.
(TIFF)

S2 Fig Confirmation of the topology of the C-terminal transmembrane domain of PfENT1. (A-C) Fluorescence (left) and phase contrast (right) images of *P*. *falciparum* parasites expressing PfENT1-S11 with cytGFP1-10 (A); PfENT1-S11 with ssGFP1-10 (B) and PfENT1-eGFP (C). (D) Immunoblots of schizont preparations from transfected parasite lines. 5 x 10^5^ schizonts were loaded per lane. Top: anti-GFP; Middle: anti-HA; Bottom: loading control, anti-BiP. Lane 1: Uninfected red bloods cells; Lane 2: Untransfected *P*. *falciparum* 3D7; Lane 3: PfENT1-S11 with cytGFP1-10; Lane 4: PfENT1-S11 with ssGFP1-10; Lane 5: PfENT1-eGFP. CytGFP1-10 is marked with a filled arrow, ssGFP1-10 is marked with an unfilled arrow, PfENT1-S11 is marked with a hash, and PfENT1-eGFP is marked with an asterisk.
(TIFF)

S3 Fig Immunofluorescence colocalisation of the ER marker BiP with the GFP signal in parasites expressing PMV-S11 with cytGFP 1–10. Red: ER marker, BiP; green: GFP. Scale bar, 2 m.
(TIFF)

S1 Text Supplementary results and discussion. (DOCX)
