# Supplementary figures and images for: Experimental Determination of the Membrane Topology of the Plasmodium Protease Plasmepsin V
Source: PLoS One. 2015 Apr 7;10(4):e0121786. doi: 10.1371/journal.pone.0121786 (PMC4388684; doi:10.1371/journal.pone.0121786)

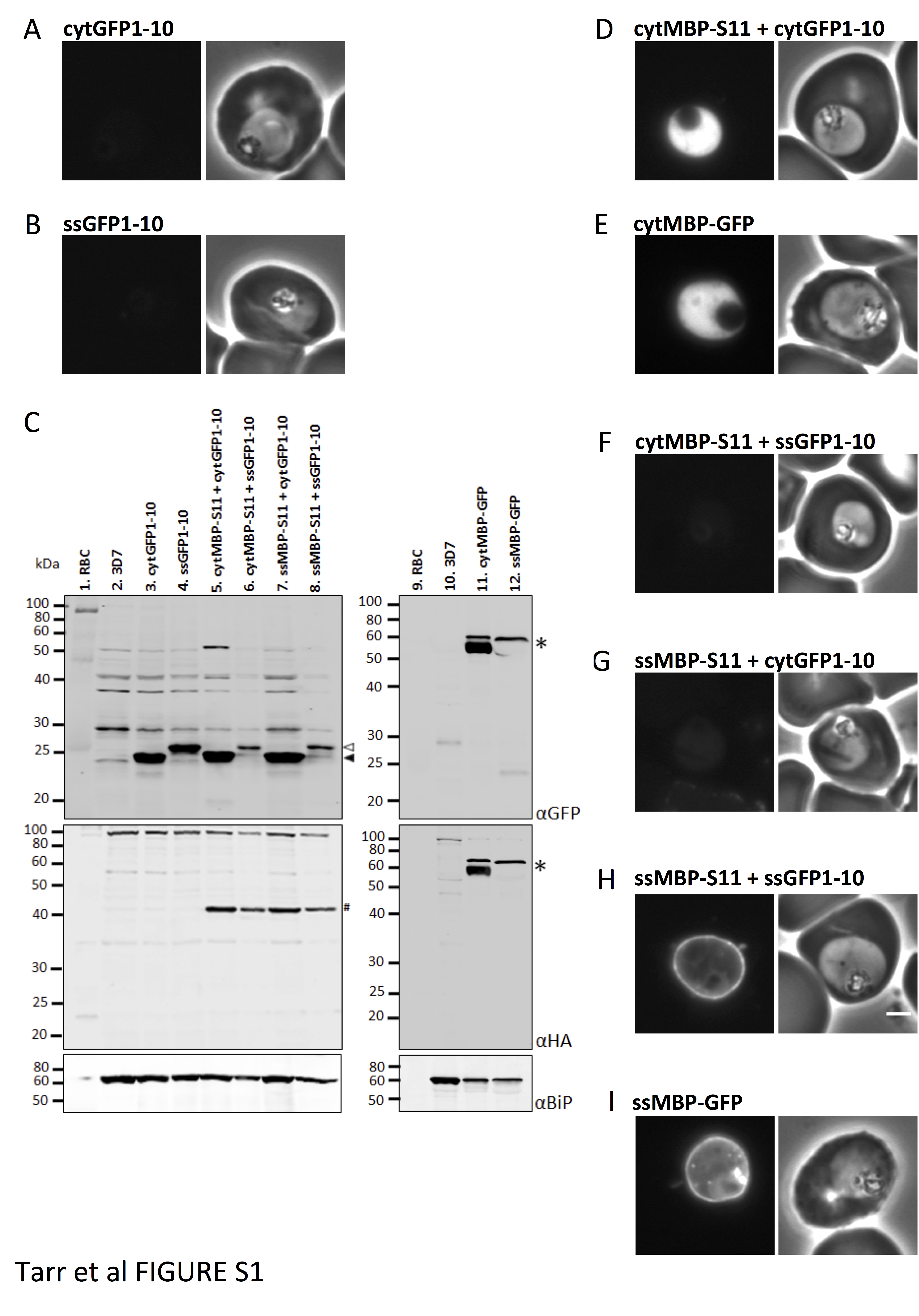

Supplement: S1 Fig — (A-B) Fluorescence (left) and phase contrast (right) images of P. falciparum parasites expressing cytGFP1-10 (A) and ssGFP1-10 (B). (C) Immunoblots of schizont preparations from transfected parasite lines. 5 x 10^5^ schizonts were loaded per lane. Top: anti-GFP; Middle: anti-HA; Bottom: loading control, anti-BiP. Lane 1: Uninfected red bloods cells; Lane 2: Untransfected P. falciparum 3D7; Lane 3: cytGFP1-10 (filled arrow); Lane 4: ssGFP1-10 (unfilled arrow); Lane 5: cytMBP-S11 with cytGFP1-10; Lane 6: cytMBP-S11 with ssGFP1-10; Lane 7: ssMBP-S11 with cytGFP1-10; Lane 8: ssMBP-S11 with ssGFP1-10; Lane 9: Uninfected red bloods cells; Lane 10: Untransfected P. falciparum 3D7; Lane 11: cytMBP-eGFP; Lane 12: ssMBP-eGFP. CytMBP-S11 and ssMBP-S11 are marked with a hash, and cytMBP-eGFP and ssMBP-eGFP are marked with an asterisk. CytMBP-eGFP migrated as a doublet. Lanes 1–4 are also depicted in Fig 1B. (D-I) Fluorescence (left) and phase contrast (right) images of P. falciparum parasites expressing cytMBP-S11 with cytGFP1-10 (D); cytMBP-eGFP (E); cytMBP-S11 with ssGFP1-10 (F); ssMBP-S11 with cytGFP1-10 (G); ssMBP-S11 with ssGFP1-10 (H) and ssMBP-eGFP (I). Scale bar, 2 m. (TIFF) [file pone.0121786.s001.tiff]

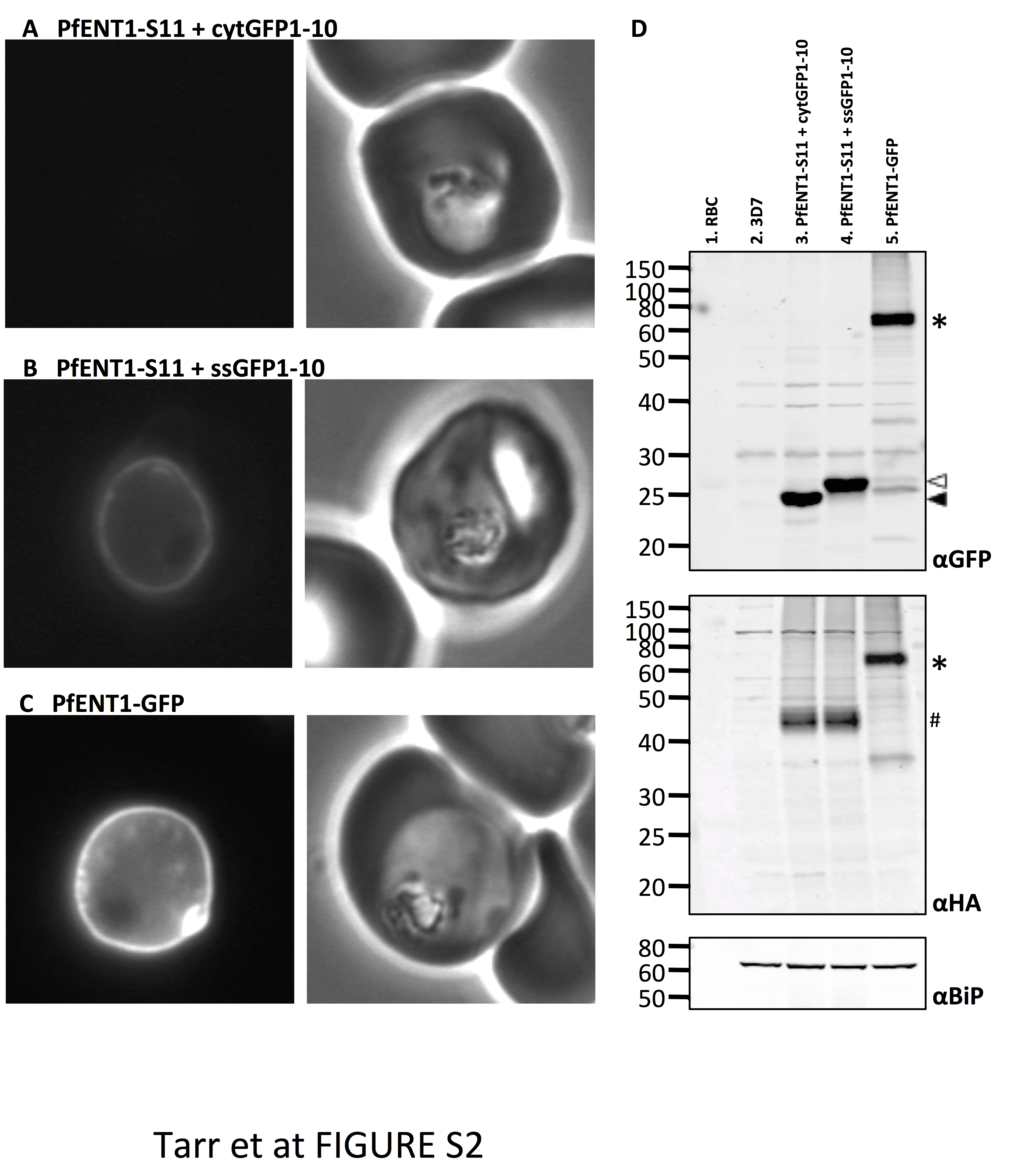

Supplement: S2 Fig — (A-C) Fluorescence (left) and phase contrast (right) images of P. falciparum parasites expressing PfENT1-S11 with cytGFP1-10 (A); PfENT1-S11 with ssGFP1-10 (B) and PfENT1-eGFP (C). (D) Immunoblots of schizont preparations from transfected parasite lines. 5 x 10^5^ schizonts were loaded per lane. Top: anti-GFP; Middle: anti-HA; Bottom: loading control, anti-BiP. Lane 1: Uninfected red bloods cells; Lane 2: Untransfected P. falciparum 3D7; Lane 3: PfENT1-S11 with cytGFP1-10; Lane 4: PfENT1-S11 with ssGFP1-10; Lane 5: PfENT1-eGFP. CytGFP1-10 is marked with a filled arrow, ssGFP1-10 is marked with an unfilled arrow, PfENT1-S11 is marked with a hash, and PfENT1-eGFP is marked with an asterisk. (TIFF) [file pone.0121786.s002.tiff]

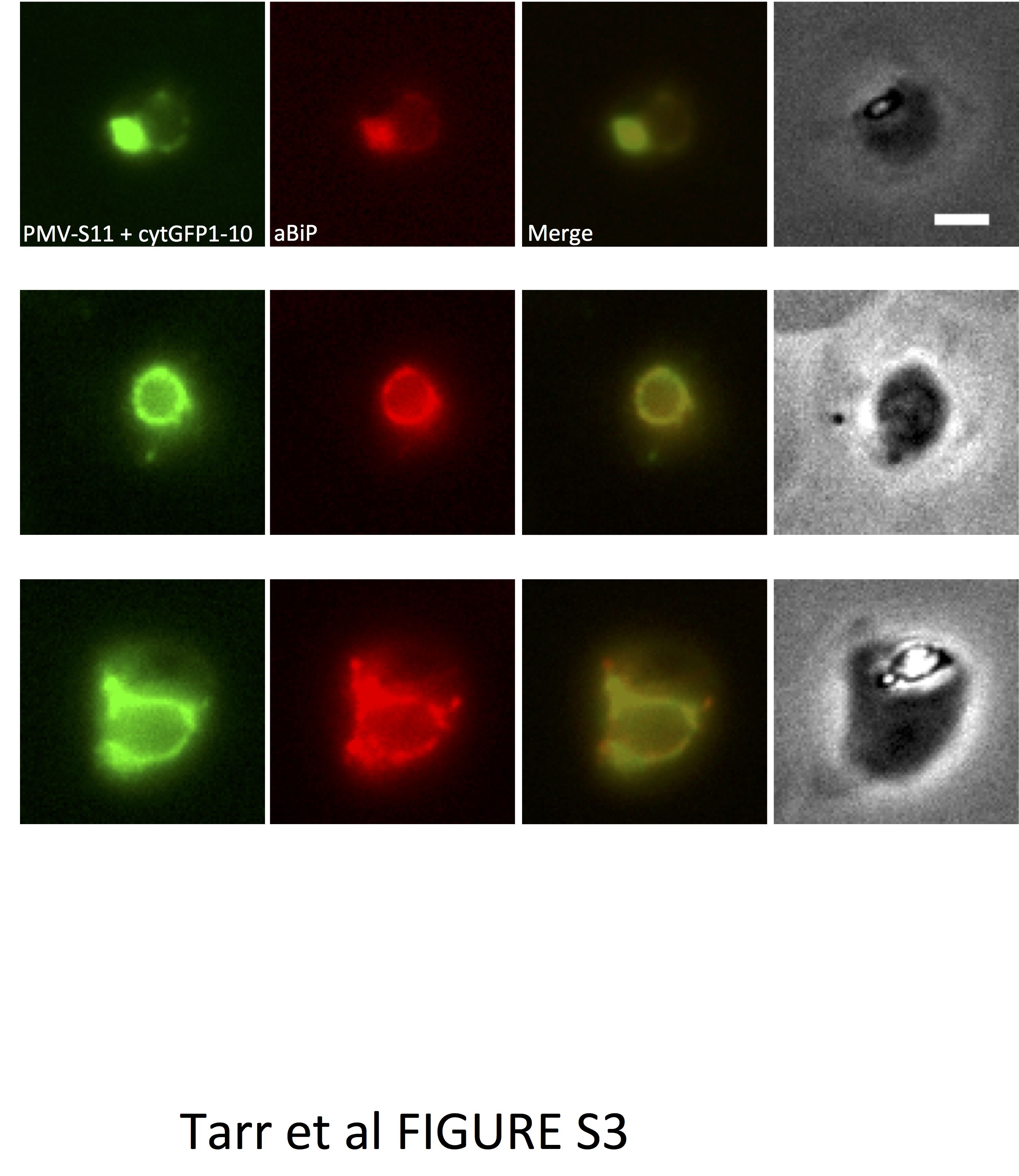

Supplement: S3 Fig — Red: ER marker, BiP; green: GFP. Scale bar, 2 m. (TIFF) [file pone.0121786.s003.tiff]
