# Supplementary material for: Experimental Determination of the Membrane Topology of the Plasmodium Protease Plasmepsin V
Source: PLoS One. 2015 Apr 7;10(4):e0121786. doi: 10.1371/journal.pone.0121786 (PMC4388684; doi:10.1371/journal.pone.0121786)
Supplement: S1 Text — (DOCX) [file pone.0121786.s004.docx]

# Supplementary results and discussion

## S11 split GFP fragments can associate to form a fluorescent complex with cytGFP1-10 and ssGFP1-10

To confirm that the cytGFP1-10 and ssGFP1-10 fragments could associate with the S11 fragment to form a fluorescent complex, we co-expressed S11 with GFP1-10 proteins. Fluorescence was dependent on the fragments being expressed in the same cellular compartments.

Bacterial maltose-binding protein (MBP) with and without its signal sequence was tagged with S11 (ssMBP-S11 and cytMBP-S11, respectively). Single HA tags were included between the MBP and S11 units for immunodetection. cytMBP-S11 and ssMBP-S11 were separately co-expressed in the parasite lines expressing cytGFP1-10 and ssGFP1-10.

When cytMBP-S11 was co-expressed with cytGFP1-10, fluorescence was observed within the parasite cytosol (S1 Fig., panel D). This distribution of fluorescence was similar to that seen for control parasites expressing cytMBP tagged with intact eGFP (cytMBP-eGFP; S1 Fig., panel E; expression of intact cytMBP-eGFP was also confirmed by immunoblotting - see S1 Fig., panel C, top and middle blots, lane 11, asterisk). However, when cytMBP-S11 was co-expressed with ssGFP1-10, no fluorescence was observed (S1 Fig., panel F). The proteins were nonetheless expressed within the parasites. CytMBP-S11 protein was detectable by immunoblotting with an anti-HA antibody as an approximately 44 kDa band in both the doubly transfected parasite lines (S1 Fig., panel C, middle blot, lanes 5 and 6, marked by a hash). In addition, the cytGFP1-10 and ssGFP1-10 proteins were detectable in these transfectants by immunoblotting of schizont preparations with an anti-GFP antibody (Fig. 1C, top blot, lane 5 filled arrow and lane 6 unfilled arrow, respectively). This confirms that when expressed in the same compartment (in this instance, the parasite cytosol), the cytGFP1-10 and cytMBP-S11 fragments associate to produce a fluorescent GFP complex.

When ssMBP-S11 was co-expressed with cytGFP1-10, no fluorescence was observed in the parasites (S1 Fig., panel G). Expression of both proteins was confirmed by immunoblotting; a band of approximately 44 kDa was detected for ssMBP-S11 with an anti-HA antibody (S1 Fig., panel C, middle blot, lane 7, marked by a hash) and the <25 kDa band for cytGFP1-10 was detected with the anti-GFP antibody (S1 Fig., panel C, top blot, lane 7, filled arrow). However, when ssMBP-S11 was co-expressed with ssGFP1-10, fluorescence was observed as a ring surrounding the parasite, suggestive of PV localisation (S1 Fig., panel H), which is in keeping with the default trafficking of signal sequence proteins through the parasite’s secretory pathway to the PV. This localisation was identical to that seen for ssMBP tagged with intact eGFP (S1 Fig., panel I). Expression of intact ssMBP-eGFP was confirmed by immunoblotting (S1 Fig., panel C, lane 12). The expression of intact ssMBP-S11 and ssGFP1-10 proteins was also verified by immunoblotting; ssMBP-S11 was detected using an anti-HA antibody (S1 Fig., panel C, middle blot, lane 8, marked by a hash) and the >25 kDa band for ssGFP1-10 was detected using an anti-GFP antibody (S1 Fig., panel C, top blot, lane 8, unfilled arrow). This again confirms that direction of the split GFP fragments to the same cellular compartment (in this case, the PV) is necessary for fluorescence.

Together, these data also confirm that for the parasite lines generated here, there is reliable separation of cytGFP1-10 and ssGFP1-10 fragments between the parasite’s cytosol and secretory pathways, respectively.

## Confirmation that split GFP can detect the topology of a transmembrane domain with its C-terminus in the secretory pathway

We have shown using split GFP that the C-terminus of Plasmepsin V resides in the parasite cytosol. We failed to detect fluorescence when S11 tagged Plasmepsin V was co-expressed with ssGFP1-10. To ensure that the ssGFP1-10 is functional, we confirmed the topology of the C-terminal transmembrane domain of *Plasmodium falciparum* Equilibrative Nucleoside Transporter 1 (PfENT1; PF3D7_1347200). PfENT1 is localised to the parasite plasma membrane and is believed to contribute to the import of purine nucleosides [[1](#_ENREF_1)]. Based on homology to human ENT1, PfENT1 is predicted to have its C-terminus within the parasite secretory pathway [[2](#_ENREF_2)], although this has not been confirmed experimentally.

In order to determine the topology of PfENT1 in the parasite plasma membrane, PfENT1 tagged at the C-terminus with a triple HA tag and the S11 strand (PfENT-S11) was expressed in *P. falciparum* lines co-expressing either cytGFP1-10 or ssGFP1-10. No fluorescence was observed when PfENT1-S11 was co-expressed with cytGFP1-10 (S2 Fig., panel A). Immunoblots of schizont preparations from parasites co-expressing cytGFP1-10 and PfENT1-S11 indicated that both proteins were expressed; the <25 kDa band for cytGFP1-10 was detectable when probed with anti-GFP (S2 Fig., panel D, top blot, lane 3, filled arrow) and a band of approximately 45 kDa was detected for PfENT1-S11 when probed with an anti-HA antibody (S2 Fig., panel D, middle blot, lane 3, hash). This mass is slightly lower than the calculated mass of 53.7 kDa; multispanning transmembrane proteins often migrate faster than their predicted mass [[3](#_ENREF_3)]. Parasites co-expressing PfENT1-S11 with ssGFP1-10, exhibited a ring of fluorescence surrounding the parasite (S2 Fig., panel B). This observation confirmed that the C-terminal S11 tag of PfENT-S11 associated with the ssGFP1-10 protein to give a fluorescent complex within the PV. Expression of intact PfENT1-S11 and ssGFP1-10 in this transfected line was verified by immunoblotting using anti-HA and anti-GFP antibodies, respectively (S2 Fig., panel D, middle blot, lane 4, marked by a hash, and top blot, lane 4, marked by an unfilled arrow, respectively). The pattern of fluorescence for these parasites was similar to that observed for PfENT1 tagged C-terminally with intact eGFP (S2 Fig., panel C). Expression of intact PfENT1-GFP was also confirmed by immunoblotting using anti-GFP and anti-HA antibodies (S2 Fig., panel D, lane 5, top and middle blots, marked by asterisks). As for PfENT1-S11, PfENT1-GFP migrated at a molecular mass that was slightly lower than the predicted mass of 95.3 kDa.

Together, these data confirm that the C-terminus of PfENT1 is located within the PV. This topology is consistent with that derived from homology modelling to hENT1 [[2](#_ENREF_2)]. These data also confirm that split GFP fragments can associate in the lumen of the secretory pathway when the S11 tag is appended to the C-terminus of a transmembrane protein.

# Supplementary references

1. Rager N, Mamoun CB, Carter NS, Goldberg DE, Ullman B. Localization of the *Plasmodium falciparum* *Pf*NT1 nucleoside transporter to the parasite plasma membrane. J Biol Chem. 2001;276(44):41095-9.

2. Parker MD, Hyde RJ, Yao SY, McRobert L, Cass CE, Young JD, et al. Identification of a nucleoside/nucleobase transporter from *Plasmodium falciparum*, a novel target for anti-malarial chemotherapy. Biochem J. 2000;349(1):67-75.

3. Rath A, Glibowicka M, Nadeau VG, Chen G, Deber CM. Detergent binding explains anomalous SDS-PAGE migration of membrane proteins. Proceedings of the National Academy of Sciences. 2009;106(6):1760-5. doi: 10.1073/pnas.0813167106.
